# Topic modeling on clinical social work notes for exploring social determinants of health factors

**DOI:** 10.1093/jamiaopen/ooad112

**Published:** 2024-01-14

**Authors:** Shenghuan Sun, Travis Zack, Christopher Y K Williams, Madhumita Sushil, Atul J Butte

**Affiliations:** Bakar Computational Health Sciences Institute, University of California, San Francisco, San Francisco, CA 94158, United States; Bakar Computational Health Sciences Institute, University of California, San Francisco, San Francisco, CA 94158, United States; Division of Hematology/Oncology, Department of Medicine, UCSF, San Francisco, CA 94143, United States; Bakar Computational Health Sciences Institute, University of California, San Francisco, San Francisco, CA 94158, United States; Bakar Computational Health Sciences Institute, University of California, San Francisco, San Francisco, CA 94158, United States; Bakar Computational Health Sciences Institute, University of California, San Francisco, San Francisco, CA 94158, United States; Center for Data-driven Insights and Innovation, University of California, Office of the President, Oakland, CA 94607, United States; Department of Pediatrics, University of California, San Francisco, San Francisco, CA 94143, United States

**Keywords:** natural language processing, topic modeling, electronic health records, social work notes, social determinants of health

## Abstract

**Objective:**

Existing research on social determinants of health (SDoH) predominantly focuses on physician notes and structured data within electronic medical records. This study posits that social work notes are an untapped, potentially rich source for SDoH information. We hypothesize that clinical notes recorded by social workers, whose role is to ameliorate social and economic factors, might provide a complementary information source of data on SDoH compared to physician notes, which primarily concentrate on medical diagnoses and treatments. We aimed to use word frequency analysis and topic modeling to identify prevalent terms and robust topics of discussion within a large cohort of social work notes including both outpatient and in-patient consultations.

**Materials and methods:**

We retrieved a diverse, deidentified corpus of 0.95 million clinical social work notes from 181 644 patients at the University of California, San Francisco. We conducted word frequency analysis related to ICD-10 chapters to identify prevalent terms within the notes. We then applied Latent Dirichlet Allocation (LDA) topic modeling analysis to characterize this corpus and identify potential topics of discussion, which was further stratified by note types and disease groups.

**Results:**

Word frequency analysis primarily identified medical-related terms associated with specific ICD10 chapters, though it also detected some subtle SDoH terms. In contrast, the LDA topic modeling analysis extracted 11 topics explicitly related to social determinants of health risk factors, such as financial status, abuse history, social support, risk of death, and mental health. The topic modeling approach effectively demonstrated variations between different types of social work notes and across patients with different types of diseases or conditions.

**Discussion:**

Our findings highlight LDA topic modeling’s effectiveness in extracting SDoH-related themes and capturing variations in social work notes, demonstrating its potential for informing targeted interventions for at-risk populations.

**Conclusion:**

Social work notes offer a wealth of unique and valuable information on an individual’s SDoH. These notes present consistent and meaningful topics of discussion that can be effectively analyzed and utilized to improve patient care and inform targeted interventions for at-risk populations.

## Introduction

Social determinants of health (SDoH) are non-medical factors that influence health outcomes, including the conditions in which people are born, grow, work, live, and age, as well as the wider set of forces and systems shaping daily life, such as economic policies, development agendas, social norms, and political systems.^1–4^ These factors contribute significantly to health disparities due to systemic disadvantages and biases.^5^^,^^6^ Systemic disadvantages refer to unequal distribution of resources and opportunities, while bias refers to unfair treatment based on social, economic, or demographic characteristics. Health inequities, which are unfair and avoidable differences in health among population groups, can arise from these determinants and warrant ethical consideration.^7^ For example, mental health during pregnancy plays a pivotal role in both the mother’s and the unborn child’s well-being.^8^ In a similar vein, lifestyle choices and living environments are intricately linked to the health outcomes of diabetes patients, with significant correlations observed.^5^ These examples illustrate how systemic disadvantages and biases contribute to health inequities, underlining the importance of addressing SDoH in medical treatments for these conditions.^5^^,^^9–11^

Social work notes written by social workers contain comprehensive information on SDoH compared to other common clinical note types documented by clinicians or medical professionals. Examples of social aspects covered in social work notes include living conditions, family support, access to transportation, employment status, and education level. While other types of notes such as nursing notes, discharge summaries, and hospital progress notes may include some SDoH-related information such as insurance status, and health-related aspect such as food and physical environment, they typically focus on specific aspects of patient care and may not provide as extensive information on SDoH as social work notes, which are written to provide a more complete view of these factors.^12–14^ However, our capacity to research sociodemographic and socioeconomic health outcomes is still quite constrained. Most assessments of SDoH are not present in structured data.^15^ Instead, much of this information is collected in unstructured notes, making the information largely inaccessible without advanced technical processing. The inability to easily extract this information limits research into the effects of SDoH on care delivery and success.

To understand the information embedded in the social work notes and to characterize specific SDoH factors covered across nearly one million notes, we explored the use of unsupervised methods for topic modeling. Topic modeling methods based on Latent Dirichlet Allocation (LDA) have been previously successful in finding hidden structures (topics) from large corpora,^16^^,^^17^ the utility of which we further explored in this study. The large collection of social work notes analyzed in this study spanned a diverse cohort of patient demographics and disease groups. This allowed us to develop a comprehensive understanding of the underlying SDoH topics from different note types for a variety of disease chapters. We explored several methods to circumvent the inherent limitations of topic modeling approaches, such as pre-determining a fixed number of clusters, intrinsic randomness, and need for human-based interpretation.

## Background and significance

Computational understanding of the free text in clinical notes is well known to be an open challenge, including the extraction of structured information from these documents.^18^ Some progress has been made in extracting SDoH factors from clinical text using named entity recognition (NER), a Natural Language Processing (NLP) method of extracting pre-defined concepts from text.^19^^,^^20^ Both machine learning-based and traditional rule-based NER have been developed and tested.^20–22^ While NER approaches have been shown to be effective, they can be time-consuming.^23^

Topic modeling methods have been widely applied for unbiased topic discovery from large collections of documents^24–26^ and have been used in the fields of social science,^27^ environmental science,^28^ political science,^29^ and in biological and medical contexts.^12^ Recent studies, such as work by Meaney et al,^12^ have begun to explore latent topics in clinical notes. However, to our knowledge, topic modeling has not been heavily used to assess corpora of social work notes for SDoH factors, likely due to the general availability of sufficiently large corpora.

Clinical social workers are licensed professionals who specialize in identifying and addressing social and environmental barriers experienced by patients. In particular, text notes documented by clinical social workers are an invaluable data resource for understanding SDoH information in patients. As such, the clinical notes written by social workers often include specific text capturing an individual’s SDoH. Yet, to date, social work notes have been a relatively under-utilized data source and have not been extensively investigated for understanding SDoH.^30^

This study aims to explore the potential of social work notes as a rich source of data on SDoH by analyzing the most meaningful social work terminology across different disease chapters and applying LDA topic modeling to identify robust topics of discussion within a large cohort of social work notes. By doing so, we seek to uncover clinically relevant SDoH information contained in these notes and their potential impact on patient and public health, demonstrating the value of social work notes in understanding SDoH factors.

## Materials and methods

### Data sources and patient demographics

This study uses the deidentified clinical notes at UCSF recorded between 2012 and 2021.^31^ The study was approved by the Institutional Review Board (IRB) of the University of California, San Francisco (UCSF; IRB #18-25163). Our cohort consists of the following demographic distribution: Gender—Male: 95 387 (52.5%), Female: 85 635 (47.1%); Race—White: 22 839 (12.6%), Black: 21 120 (11.6%), Asian: 47 723 (26.3%), Native American: 14 813 (8.2%), Other: 75 149 (41.4%); Age—Median: 33 years (Range: 12-58); Ethnicity—Hispanic: 41 386 (22.8%), Non-Hispanic: 128 018 (70.5%).

### Data preprocessing

**Figure 1.**
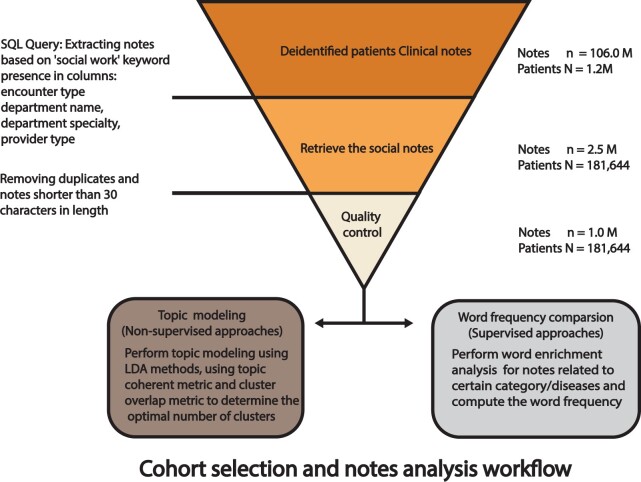
Retrieval of clinical social work notes for the study. The social work notes from the UCSF Information Commons between 2012 and 2021 were initially retrieved. Notes that were duplicated or extremely short were excluded, which resulted in a corpus of 0.95 million notes. Later, the notes were analyzed using 2 methods: word frequency calculation (Bottom Left) and topic modeling (Bottom Right). Later, the word frequency was compared between different disease chapters. For topic modeling, Latent Dirichlet Allocation was used to identify the topics in individual social work notes. Topic coherence metric and Jaccard distance were implemented to decide the optimal clustering results.

We initiated our research by collecting clinical notes from a de-identified dataset, specifically selecting those entries where the metadata contained the term “social”—case-insensitive—within the encounter type, department name, specialty, or provider type, thus designating these as “social work notes.” From the extensive corpus of 106 million notes representing 1.2 million patients, this focused query yielded 2.5 million social work notes attributed to 181 644 unique patients. To ensure the quality and relevance of our data, we excluded notes under 30 characters, anticipating they would not provide substantial content. Duplicate notes were also removed to eliminate redundancy and decrease computational demands. Following this stringent quality control process, we distilled the dataset down to 1 million notes corresponding to the same 181 644 patients, which formed the basis for our downstream topic modeling analysis, as depicted in Figure 1.

### Topic modeling with LDA analysis

While word frequency calculations can provide preliminary insights about term relevance, this view is too limited to understand what broader topics may be contained within social work notes. In contrast, topic modeling is a field of unsupervised learning that learns statistical associations between words or groups of words to identify “topics”: clusters of words that tend to co-occur within the same document.

LDA is a generative probabilistic model, which assumes that each document is a combination of a few different topics, and that each word’s presence can be attributed to particular topics in the document. The result is a list of clusters, each of which contains a collection of distinct words. The combination of words in a cluster can be used for topic model interpretation. Python package *gensim* was used for the implementation.^32^ We used *gensim.models.ldamodel.LdaModel* for the actual analysis. The core estimation code is based on Hoffman et al.^33^

Python package *nltk* was used. As a preprocessing step, English language stop words and special characters including “\t,” “\n,” “\s” were removed from note text. The resulting text from all social work notes were vectorized and topics were inferred with the LDA algorithm. In addition to the analysis on the complete cohort of social work notes, in order to investigate the topic distribution across specific social work note categories, we additionally analyzed the 4 largest categories of social work notes: *Progress Notes*, *Interdisciplinary*, *Telephone encounters*, and *Group Notes*. We also extended the investigation to social work note subsets across 10 ICD-10 disease chapters. These subsets were determined by investigating encounter-specific ICD-10 diagnostic codes. The common stop words were also excluded, using *stopwords.words(“english”)* from *nltk* package.^34^ To overcome the inherent stochasticity of topic modeling approaches and ensure the reliability of our findings, we ran 5 independent modeling analyses for each category of notes. This allowed us to capture consistent patterns and topics across different iterations, increasing our confidence in the identified topics and their relevance to the respective disease groups. Another critical step in LDA topic modeling was determining the optimal cluster number, which is further discussed in the next subsection. Furthermore, when extending the analysis to different note types, we labeled the inferred topics using heuristics described further.

### Determining the optimal number of topics for notes

One of the most important hyper-parameters for LDA analysis is the number of topics K. Generally, if K is chosen to be too small, the model will lack the capacity to provide a holistic summary of complex document collections; and returned topical vectors may combine semantically unrelated words/tokens.^35^ Conversely, if K is chosen to be too large, the returned topical vectors may be redundant, and a parsimonious explanation of a complex phenomenon may not be achieved. We used 2 evaluation metrics, topic coherence^36^^,^^37^ and topic similarity,^38^ to systematically determine the optimal number of clusters. Topic Coherence (C) quantifies the score of a single topic by measuring the degree of semantic similarity between high-scoring words in the topic.^39^ The measure helps distinguish between topics that are semantically interpretable and those that are artifacts of statistical inference. The coherence metric we compute is based on a sliding window, one-set segmentation of the top words and an indirect confirmation measure that uses normalized pointwise mutual information and the cosine similarity.^37^ Similarly, topic similarity (S) measures how similar 2 clusters are considering the words contained in the topics. The lower the values are, the less redundant the topic distribution is. For quantifying topic similarity, we use Jaccard similarity.^38^ Furthermore, there are alternative ways to evaluate the quality of topic discovery, such as assessing “topic diversity.”^40^ Considering these evaluation metrics in future work may provide further insights into the performance of our methods.

An ideal solution would have a high topic coherence and low similarity metric. To decide the optimal number of clusters, for each analysis, we ran the LDA analysis with the number of clusters K ranging from 10 to 50, simultaneously computing C and S scores. The number having the *i_th_* highest C value, *j_th_* smallest S value, and the minimum *i* + *j* among all runs was selected as the final number of topics (Figure S1A). We found that the best cluster number for analyzing the entire notes repository was 17.

### Topic modeling per notes with certain type

In order to investigate the topic distribution across specific note categories, we applied topic modeling on the 4 largest categories of social work notes: *Progress Notes*, *Interdisciplinary*, *Telephone encounters*, and *Group Notes*. This approach allowed us to gain insights into the prevalence of certain topics within these major categories and assess their potential impact on the overall topic modeling results. We used the same pipeline for identifying the optimal number of clusters as described earlier in the Materials and methods section. To ensure robustness in our results, given the inherent randomness of the LDA method, we conducted each analysis across 5 different iterations for every category. This approach allowed us to capture a broader range of variability, thereby increasing the reliability of our findings. The results from these 5 iterations were then pooled together. This pooling strategy was instrumental in developing a well-grounded heuristic for labeling the topic clusters, ensuring our results were reflective of consistent patterns observed across all iterations, rather than being influenced by any single run’s anomalies.

In our analysis, we determined that the optimal number of clusters for most of the analyses we conducted is ∼20. This balances the trade-off between coherence and similarity metrics, ensuring that we obtain semantically interpretable and non-redundant topic clusters, which provide meaningful insights into the underlying document collection. Consequently, we used 20 clusters for the majority of our note analyses, including those focused on note subtypes or disease chapters. However, we found that the best cluster number for analyzing the entire notes repository was 17, so we utilized 17 clusters for the topic modeling of all notes combined (see previous session).

### Topic labeling heuristics

Apart from labeling topics determined from the entire cohort of social work notes, our analysis screened 20 topic clusters (determined experimentally; see Results) for all 14 categories of notes (10 disease chapters and 4 social work note types) for 5 independent runs (to reduce stochasticity), thereby resulting in 1400 topic clusters that required further labeling. To assign labels to all 1400 topics, we developed a heuristic to automatically assign topic labels for subsequent analyses, the details of which are discussed next.

We first constructed the dictionary of topic names and the corresponding words by manually analyzing the topic modeling results for one run on the complete corpus of 0.95 million social work notes at UCSF. Then we expanded the individual topic clusters by first retrieving 20 most similar words to the words comprising topic clusters based on the cosine similarity of their word embeddings.^41^ Any words that were not relevant to the topic label, as determined through manual review, were not considered further. The final dictionary of topic labels and the set of words used to label the topics is shown in Table 2. In our approach, we automatically assigned topic labels to individual word clusters by calculating the intersection over union (IOU) ratio for the words in a cluster. This enabled us to assign labels to all 1400 topic clusters from our analysis. The details can be found in the pseudo-code below. To address your professor’s concerns, we used the IOU of word frequencies within each cluster. We assigned the label with the maximum IOU, but only if there was an overlap of at least 2 words. If none of the topics met this criterion, we did not assign a topic to the word cluster.

*Heuristics of automatic assigning topic names for the individual topic cluster*

***Begin:***

*Construct the dictionary of topic names and the words comprising this topic;*

*Expand the individual topic cluster space;*

*> Enrichment[k|h] means the frequency of words belong to topic h for word cluster k*

> ∩ means intersect, ∪ means union

***For*** *each iteration of the topic modeling results* ***do:***

 ***For****every word cluster k* ***do:***

  ***For****topic name h, corresponding word set* ***in topic****dictionary* ***do:***

   overlap = word cluster k ∩ topic h

  total = word cluster k ∪ topic h

   *Enrichment[k|h]* ***=****overlap/total*

  *The topic assigned to word cluster k = max([Enrichment[k|h] for h in H])*

***End***

Code for the paper is available on https://github.com/ShenghuanSun/LDA_TM

### Word frequency calculation

To perform a preliminary investigation of disease-specific features in the social work notes, 10 disease chapters were identified with ICD-10 codes: (1) Diseases of the nervous system (G00-G99), (2) Diseases of the circulatory system (I00-I99), (3) Diseases of the respiratory system (J00-J99), (4) Diseases of the digestive system (K00-K95), (5) Diseases of the musculoskeletal system and connective tissue (M00-M99), (6) Diseases of the genitourinary system (N00-N99), (7) Pregnancy, childbirth and the puerperium (O00-O9A), (8) Congenital malformations, deformations and chromosomal abnormalities (Q00-Q99) (9) Neoplasms (C00-D49), and (10) Diseases of the blood and blood-forming organs and certain disorders involving the immune mechanism (D50-D89).

Chi-squared statistics was used to compare the frequency of words across different note categories (χ^2^ function from *sklearn.feature* selection was used to this end). After ranking the *P* values and removing stop words, the top 5 potential meaningful words were visualized by the word frequency calculation. Python package *scikit-learn* was used to conduct the analysis.^42^ To embed and tokenize the unstructured notes, *text*. *CountVectorizer* function from *sklearn.feature* extraction package was used.

## Results

We retrieved a total of 0.95 million de-identified clinical social work notes generated between 2012 and 2021 (see Materials and methods) from our UCSF Information Commons^31^ (Figure 1). The majority of notes were classified as Progress Notes, Interdisciplinary Notes, or Telephone Encounter Notes; other note categories included Patient Instructions, Group Note, Letter, which comprised fewer than 5% each. These notes covered 181 644 patients of which 95387 (52.5%) were female. The median age of these patients was 33 years. Among them, 69 211 patients had only one note; 65 100 patients had between 2 and 5 notes, and 47 333 patients had more than 5 notes (Table S1, Figure S2B). The demographics distribution is presented in Table 1. No demographic feature was statistically associated with the number of notes for each patient (Table S1).

**Table 1.** Topic modeling results for all social work notes.

Clusters	Key words
1	goal, anxiety, problem, term, depression, mood, therapy, symptom, long, treatment
2	recommendation, wife, education, treatment, patient, form, appearance, ongoing, advocate, trauma
3	hospital, self, day, pain, other, connection, recent, feeling, side, number
4	mother, father, family, room, information, nurse, source, concrete, control, instruction
5	session, consultation, telehealth, location, time, tool, objective, parking, other, treatment
6	parent, family, school, child, sister, support, place, year, well, initial
7	group, intervention, patient, discussion, response, time, summary, progress, participant, skill
8	risk, chronic, thought, normal, imminent, status, testing, intervention, speech, suicide
9	client, health, service, caregiver, mental, therapist, therapy, behavioral, individual, group
10	well, when, time, week, also, able, state, more, friend, very
11	social, service, support, family, assessment, medical, time, note, concern, ongoing,
12	care, home, plan, phone, contact, work, information, resource, call, support
13	time, clinician, name, date, code, behavior, risk, number, plan, provider
14	history, child, other, factor, current, none, substance, abuse, psychiatric, year
15	donor, donation, potential, employment, understanding, risk, decision, independent, process, care
16	night, morning, hour, sleep, house, already, less, past, aggressive, evening
17	transplant, medication, post, support, health, insurance, husband, psychosocial, message, history

Each row is an inferred topic, which is composed of 10 words.

**Table 2.** Topic assignment heuristic.

Topics	Keywords
Mental health	mental, depression, anxiety, mood, psychological, physical, cognitive, emotional, mind, psychiatric
Family	family, parent, father, mother, child, children, sister, parents, relatives, clan, childhood, friends
Consultation/appointment	appointment, consultation, consult, questionnaire, question, advice, biographical, wikipedia, relevant, questions, know, documentation
Group session	group, intervention, session, interpers, community, class, organization, together, part, organization
Risk of death	suicide, suicidal, risk, crisis, homicide, murder, commit, bombing, murdered, murders, bomber, killing, convicted, victims
Clinician/hospital/medication	patient, medication, hospital, medical, clinic, clinician, treatment, therapy, surgery, symptoms, patients, drugs, diagnosis, treatments, prescribed
Living condition/lifestyle	shelter, housing, house, living, sleep, bedtime, building, buildings, urban, employment, suburban, campus, acres
Social support	social, service, support, referral, recommendation, recommend, worker, resource, supports, provide, supporting, supported, allow, providing, assistance, benefit, help
TelephoneEcounter/online communication	telehealth, phone, call, video, telephone, mobile, wireless, gsm, cellular, dial, email, calling, networks, calls, messages, telephones, internet
Abuse history	abuse, history, addiction, alcohol, drugs, allegations, victim, violence, sexual, rape, dependence
Insurance/income	insurance, income, coverage, financial, contracts, banking, finance, liability, private, pay

The words in the *Keywords* column are the representative words used to define the topics.

In addition to analyzing the number of notes, we were also interested in exploring the medical conditions associated with patients who received social work notes. This aspect can provide valuable insights into the factors contributing to the need for social work intervention. To investigate this, we collected the ICD-10 codes for the encounters during which social work notes were recorded for the patients. These ICD-10 codes were then mapped at the chapter level.^33^ The 3 most frequent ICD-10 chapters found to be associated with a social work note were “Mental, Behavioral and Neurodevelopmental disorders,” “Factors influencing health status and contact with health services,” and “Symptoms, signs and abnormal clinical and laboratory findings, not elsewhere classified” (Table S2).

### Using LDA to extract topics in social work notes

Looking at the word components of each topic (Table 1), we discovered a few diverse clusters that cover many different social aspects of patients including social service (Topic 11), abuse history (Topic 14), phone call/online communications (Topic 12), living condition/lifestyle (Topic 16), risk of death (Topic 8), group session (Topic 7), consultation/appointment (Topic 5), family (Topic 4, 6), and mental health (Topic 1). Many of these topics are consistent with topics covering SDoH; most importantly, most of the information potentially conveyed through these topics are absent in the structured data. Of note, in our parameter exploration, we found that increasing the number of clusters can lead to additional recognizable topics, such as food availability (data not shown), although we also obtain redundant topics.

### Topic modeling on specific note categories

**Figure 2.**
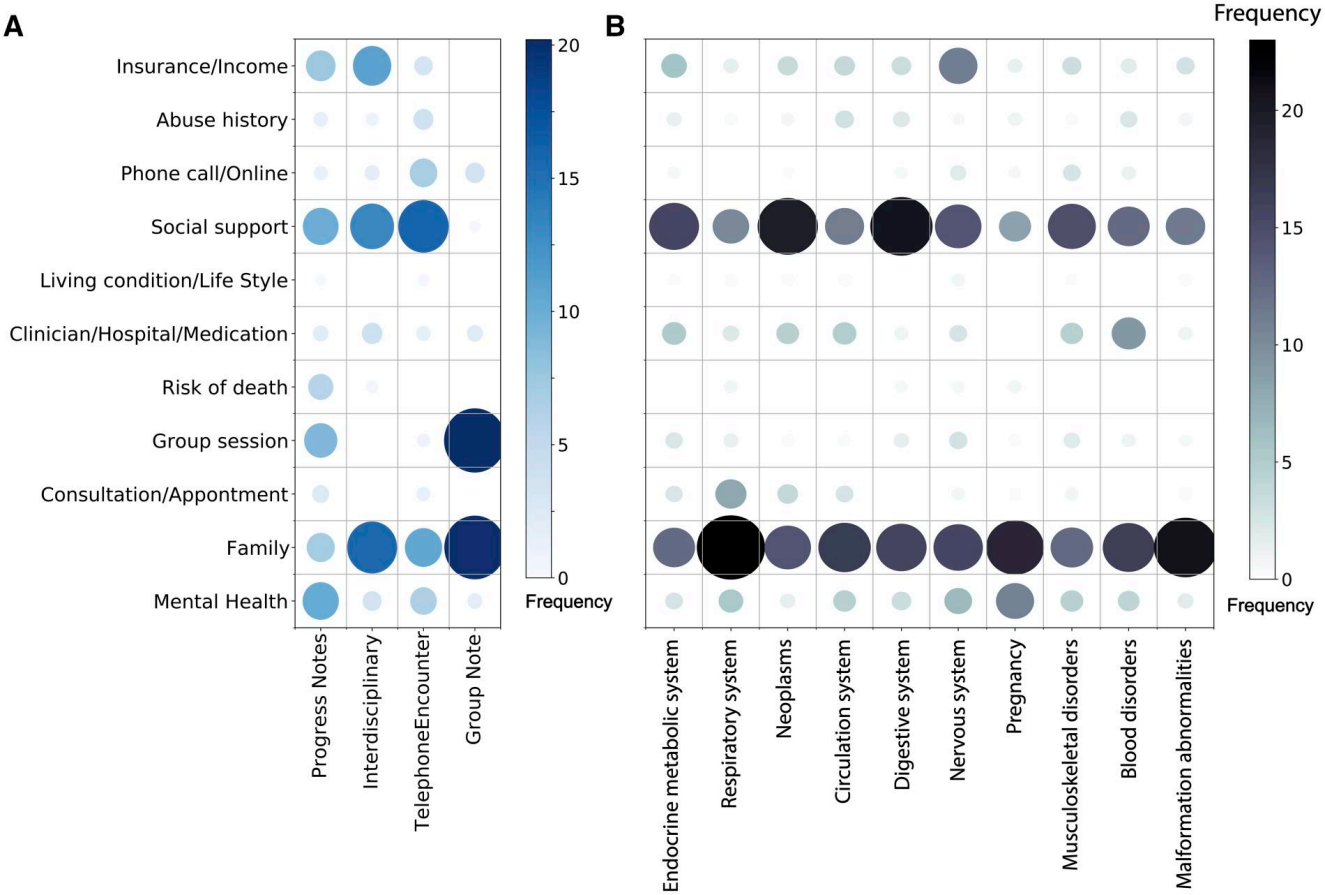
Topic proportion comparison for different categories. (A) Topic proportion comparison for different note types. (B) Topic proportion comparison for different disease chapters. Size and color of the circle represent proportion of each topic.

Analyzing the topics appearance in each note subtype, we found that social work notes in the Progress Notes category contained a higher percentage of clinically related topics, such as Mental Health (4.32%) and Clinician/Hospital/Medication-related information (8.40%), along with a smaller proportion of SDoH-related topics like Insurance/Income, Abuse history, Social support (10.46%), and Family (6.29%). Compared to Progress Notes, Telephone Encounter notes contained a larger proportion of topics related to Insurance/Income (3.93%), Phone call/Online (7.47%), Social support (11.56%), and Family (8.08%). Interestingly, telephone encounter notes lacked information about the Risk of death (0%), which may be because the discussions on this topic are not appropriate for telephone encounters. Furthermore, Group Notes, which are the notes taken during group therapy, describe the group’s progress and dynamics. As expected, Group Notes have a more uneven topic category distribution, with a higher percentage of Group session (24.69%) and Phone call/Online (12.71%) related topics (Figure 2A).

We also applied LDA analysis to the social work notes associated with 10 ICD-10 chapters described earlier (Figure 2B). We observed that most diseases have a similar topic proportion distribution, for example, most of them are enriched for Social support and Family topics. In particular, Social support is highly represented in notes related to Neoplasms (21.51%) and Diseases of the digestive system (22.47%). Family topics are also frequently mentioned in notes associated with Diseases of the nervous system (23.31%), Pregnancy, childbirth, and the puerperium (20.1%), and Congenital malformations, deformations, and chromosomal abnormalities (21.43%). However, some differences were identified between the ICD-10 chapters. Notes associated with disorders of mental health and pregnancy contain a higher percentage of SDoH topics on mental health, as would be expected. Mental health topics are more frequently mentioned in clinical notes around pregnancy than even in nervous system disorders. Interestingly, the Family topic area was often mentioned in notes associated with congenital malformation abnormalities. In summary, the analysis demonstrated both the commonness and uniqueness of topics around SDoH covered across the various diseases and conditions which afflict patients.

### Word frequency on individual disease

**Figure 3.**
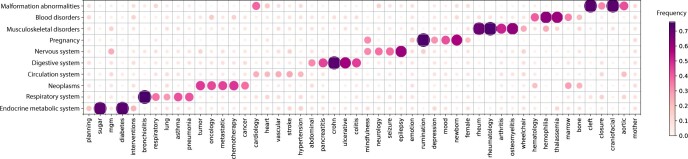
Word frequency calculation for social work notes associated with each ICD-10 chapter. The proportion of the words in social work notes associated with each ICD-10 chapter is shown by the heatmap.

In addition to performing topic modeling on social work notes associated with 10 ICD-10 chapters, we also conducted a word frequency analysis. This analysis highlighted that note from each ICD-10 chapter contained both disease-specific terms and a limited number of disease-specific SDoH topics. For instance, notes from patients with neoplasms frequently mentioned terms like “oncology,” “chemotherapy,” and “tumor,” while those associated with musculoskeletal disorders often included words such as “arthritis” and “rheumatology.” In addition to these disease-specific words, there were observable patterns in the prevalence of certain SDoH-related terms. Words like “mindfulness” appeared predominantly in chapters on Pregnancy and the Nervous System, and “wheelchair” was a recurrent term in Musculoskeletal disorders. Notably, conditions related to pregnancy showed a significant presence of mental health topics, indicating a frequent assessment of this aspect in social work notes for pregnancy care (Figure 3).

Overall, the word frequency analysis serves as a complementary tool to topic modeling. While topic modeling is adept at uncovering general patterns, predominantly SDoH topics, in social work notes, word frequency analysis, with its focused approach, tends to reveal features specific to particular diseases, especially when comparing different ICD-10 chapters.

## Discussion

We used an unsupervised topic modeling method called LDA modeling on our corpus of 0.95 million de-identified clinical social work notes. We showed that topic modeling can be used to (1) extract the hidden themes from this huge corpus of clinical notes and identify the critical information embedded in the notes, namely SDoH factors; and (2) calculate the proportion of each theme across different subsets of the note corpus and systemically characterize notes of different types. Using simple term frequency methods on this large corpus, we found that specific SDoH terms tend to be enriched in notes from patients within different disease categories, including wheelchair for patients with musculoskeletal disorders and depression for patients with pregnancy diagnoses, suggesting that these populations may be more at risk for these SDoH features.

We extracted several concrete SDoH-related topics, thus providing insight into the information that may be extracted from these corpora for facilitating future work around understanding how these topics correlate with health outcomes. During our comparison of notes of different subtypes, we found that the topic distribution of notes for specific types of diseases contains similar information but showed different levels of enrichment, representing the unique features of each disease set. As one of many examples, our analysis shows how mental health issues are frequently documented around pregnancy (Figure 2B). This type of information can help us better understand the social determinants of most concern to patients when interacting with the health system.

The specific topics identified in our study were in line with findings from a previous publication.^12^ This recent research extracted information on physical, mental, and social health by applying the non-negative matrix factorization (NMF) topic modeling method to 382 666 primary care clinical notes. However, that study exclusively examined physician-generated notes, whereas our focus was on social work notes, enabling us to uncover a broader range of SDoH topics. In our paper, we identified several additional topics, including but not limited to Living Condition/Lifestyle, Family, Risk of Death, and Abuse History.

Our research has several potential use cases. First, it aids computational sociology and epidemiology studies by identifying key factors that influence health outcomes. This extraction process lays the groundwork for in-depth analysis within these fields. Second, the findings from computational analyses can substantiate policy decisions. By providing empirical evidence, these findings can guide regulations and interventions aimed at health equity. Lastly, for participating healthcare providers, these extracted SDoH factors offer insights for effective resource allocation, particularly in supporting vulnerable groups. Overall, understanding the distribution of SDoH topics in patient records is crucial for developing targeted interventions and preventive strategies, aimed at addressing the root causes of health disparities.

Our study has several strengths. We performed analysis on a large corpus of notes, which to our knowledge, is the largest social work notes dataset to be used in a similar study. Instead of focusing on a single disease category or specific medical topic, we aimed at comprehensively finding the potential SDoH topics in all types of clinical social notes for a variety of diseases. Furthermore, to obtain a thorough understanding of the information embedded in social worker notes and capture the richness and complexity of the rhetoric in these notes, we conducted complementary analyses: a word frequency enrichment analysis allowed us to identify specific terms more frequently associated with particular ICD-10 chapters, which demonstrated the prevalence of disease-related terms in social work notes, providing a more granular view of the data. Second, the use of LDA allowed us to identify broader topics of increased relevance in these disease groups. It helped us uncover patterns related to SDoH, offering a higher-level perspective on the data.

Recognizing the intrinsic instability of LDA topic modeling methods, we enhanced the robustness of our results by independently searching for optimal hyperparameters to predefine topic numbers. Additionally, we ensured reliability by conducting each analysis across 5 iterations for every category (see Materials and methods). However, it is possible to still obtain different topic clusters with a different set of hyperparameters. Moreover, other topic modeling algorithms, such as NMF^12^^,^^43^ and BERTopic,^44^ could be explored to compare their performance and suitability for our specific task. In addition, we developed topic labeling heuristics that allow us to assign topics to the individual clusters. However, the heuristics may not cover all topic-related keywords, and in the future, it may be interesting to revisit our heuristic to expand upon the topic clusters further to make them more generalizable. State-of-the-art large language models like ChatGPT offer significant potential for improving our pipeline, particularly in the nuanced task of assigning topic labels.^45–47^ With effective prompt engineering, these models could systematically extract patterns from social work notes, enhancing the depth and accuracy of our statistical analyses, and potentially uncovering new insights in SDoH. We also exclusively utilized ICD-10 codes, acknowledging the prospective merit of incorporating ICD-9 in future research. Another limitation of our study is the lack of structured Electronic Health Record (EHR) data for recording comorbidities, insurance, and living status. These factors are relevant to SDoH and could provide valuable insights into the relationships between health outcomes and social determinants. The absence of such data may limit our ability to fully capture the complex interplay of these factors and their effects on health. Finally, we did not explicitly exclude negations or the lengthy expression, as they still contribute to the overall discussion of certain topics. However, we acknowledge that the consideration of negation is crucial for a more nuanced understanding of the information contained in clinical notes, and for more accurate analysis of the semantic meaning of the identified topics.

Our study opens pathways for several key areas of future research. For data scientists and computational researchers, future research should focus on combining these identified themes with predictive modeling techniques to assess their correlation with future health outcomes. This integration would not only validate the relevance of the identified SDoH themes but also provide a more holistic understanding of patient care dynamics and health outcomes. For healthcare practitioners, the challenge lies in integrating SDoH insights into patient care and public health policies. This demands not only an understanding of clinical informatics but also an insight into health policy and administration. Collaborating with experts in these fields could lead to developing actionable strategies that utilize our findings to improve healthcare delivery and policy decisions.

## Conclusion

Social work notes contain rich and unique information about SDoH factors, frequently only recorded in text notes. SDoH factors are critical for analyzing health outcomes, and this study identified detailed categories of SDoH information covered by social work notes. Furthermore, the study demonstrated that different categories of notes emphasize different aspects of SDoH, despite belonging to social work consultations. The findings from this study would form a basis of potential future research questions around this utilizing SDoH to uncover health disparities and SDoH-associated disease trajectories, as well as methods to extract comprehensive SDoH-related information from clinical notes.

## Supplementary Material

ooad112_Supplementary_Data

## Data Availability

The data that support the findings of this study are available from the Information Commons platform at UCSF, but restrictions apply to the availability of these data, which were used under license for the current study, and so are not publicly available. Data are however available from the authors upon reasonable request and with permission of UCSF.
