# Supplementary material for: Topic modeling on clinical social work notes for exploring social determinants of health factors
Source: JAMIA Open. 2024 Jan 14;7(1):ooad112. doi: 10.1093/jamiaopen/ooad112 (PMC10788143; doi:10.1093/jamiaopen/ooad112)
Supplement: ooad112_Supplementary_Data [file ooad112_supplementary_data.docx]

SUPPLEMENTARY MATERIALS

**
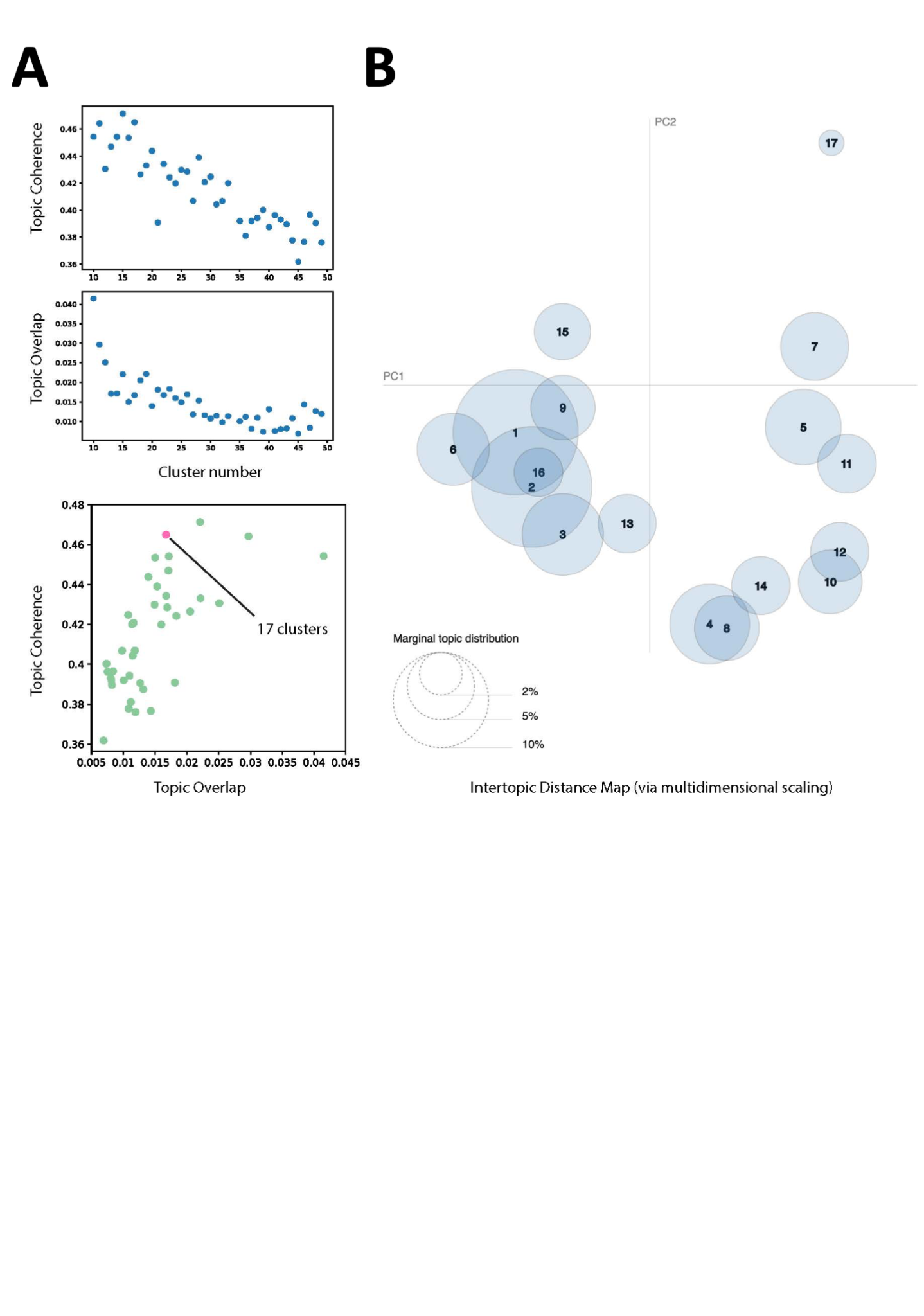
**

**S. Figure 1. Topic modeling clustering on whole social work notes.** A. Pipeline for determining the optimal number of clusters for the LDA method. The top: the plot of cluster number versus the topic coherence metric; The middle: the plot of the cluster number versus the topic overlap metric (measured by jaccard similarity metric); The bottom: the plot of the topic overlap metric versus the topic coherence metric. The number of clusters is chosen as 17 because it has the lowest topic overlap metric value while having the highest topic coherence metric value (see **Methods**) B. Inter-topic distance mapping for the individual cluster. Each circle represents an inferred topic. The coordinates for each circle correspond to the first two Principal components. The radius size indicates the frequency of topic existence on each note.


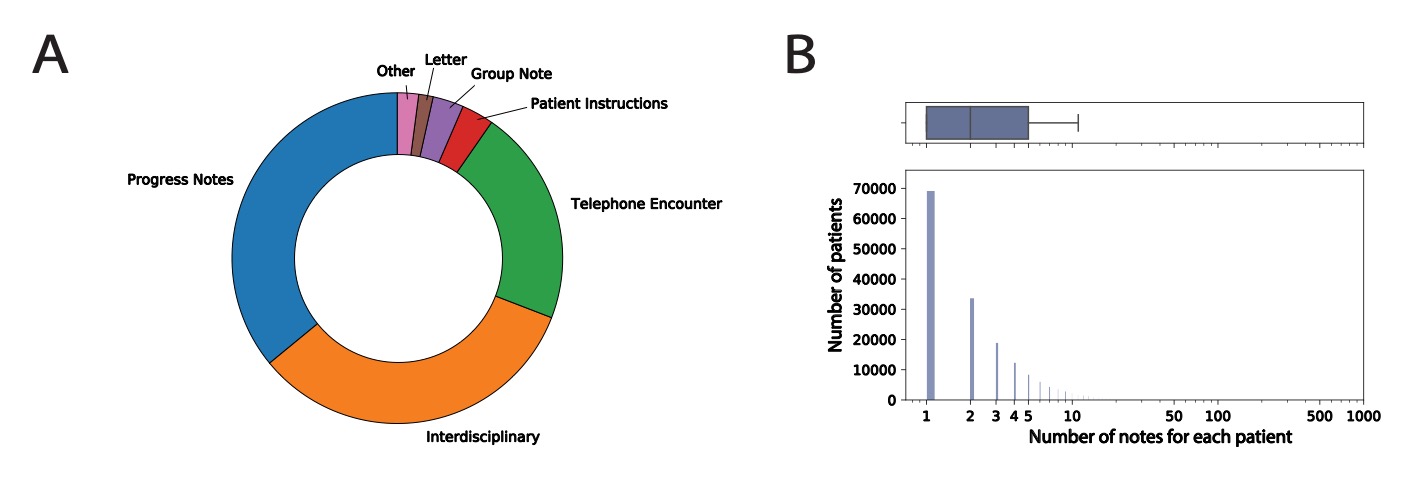


**S. Figure 2. Data exploration on social work notes.** A. Pie chart showing the proportions of patients in different categories. B. Boxplot and histogram showing the number of notes for the individual patients. The scale of x-axis is log10-transformed. The mode, mean, and median are 1, 5.8, and 2.

**S. Table 1:** Descriptive statistics for clinical social work notes corpus and contributing patient samples. Few Notes: Number of notes <= 1; Several Notes: 2<=Number of notes < 5; Many Notes: Number of notes >= 5.

|  | **Few Notes**  **(N=69211)** | **Several Notes**  **(N=65100)** | **Many Notes**  **(N=47333)** | **Overall**  **(N=181644)** |
| --- | --- | --- | --- | --- |
| **Sex** |  |  |  |  |
| Female | 36372 (52.6%) | 34285 (52.7%) | 24730 (52.2%) | 95387 (52.5%) |
| Male | 32608 (47.1%) | 30626 (47.0%) | 22401 (47.3%) | 85635 (47.1%) |
| Unknown | 231 (0.3%) | 189 (0.3%) | 202 (0.4%) | 622 (0.3%) |
| **Ethnicity** |  |  |  |  |
| Hispanic/Latino | 14891 (21.5%) | 14451 (22.2%) | 12044 (25.4%) | 41386 (22.8%) |
| Not Hispanic/Latino | 48758 (70.4%) | 46011 (70.7%) | 33249 (70.2%) | 128018 (70.5%) |
| Unknown | 5562 (8.0%) | 4638 (7.1%) | 2040 (4.3%) | 12240 (6.7%) |
| **Race** |  |  |  |  |
| Asian | 8651 (12.5%) | 8578 (13.2%) | 5610 (11.9%) | 22839 (12.6%) |
| Black/African | 7153 (10.3%) | 7148 (11.0%) | 6819 (14.4%) | 21120 (11.6%) |
| Other | 17594 (25.4%) | 16922 (26.0%) | 13207 (27.9%) | 47723 (26.3%) |
| Unknown | 6683 (9.7%) | 5493 (8.4%) | 2637 (5.6%) | 14813 (8.2%) |
| White | 29130 (42.1%) | 26959 (41.4%) | 19060 (40.3%) | 75149 (41.4%) |
| **Age** |  |  |  |  |
| median (Q1-Q3) | 32 (11 - 58) | 35 (13 - 59) | 30 (10 - 57) | 33 (12 - 58) |
| Missing | 111 (0.2%) | 135 (0.2%) | 175 (0.4%) | 421 (0.2%) |

**S. Table 2.** Most frequent ICD-10 codes for patients with social work notes

| **ICD10 code** | **Diagnosis** | **Notes** | **Patients** |
| --- | --- | --- | --- |
| **F01-F99** | **Mental, Behavioral and Neurodevelopmental disorders** | **106348** | **15388** |
| **Z00-Z99** | **Factors influencing health status and contact with health services** | **36399** | **25995** |
| **R00-R99** | **Symptoms, signs and abnormal clinical and laboratory findings, not elsewhere classified** | **25011** | **18820** |
| **E00-E89** | **Endocrine, nutritional and metabolic diseases** | **13307** | **8488** |
| **S00-T88** | **Injury, poisoning and certain other consequences of external causes** | **10508** | **5092** |
| **I00-I99** | **Diseases of the circulatory system** | **9832** | **8450** |
| **K00-K95** | **Diseases of the digestive system** | **7711** | **6267** |
| **G00-G99** | **Diseases of the nervous system** | **7589** | **6187** |
| **O00-O9A** | **Pregnancy, childbirth and the puerperium** | **7370** | **5628** |
| **D50-D89** | **Diseases of the blood and blood-forming organs and certain disorders involving the immune mechanism** | **6289** | **4598** |
| **Q00-Q99** | **Congenital malformations, deformations and chromosomal abnormalities** | **6041** | **4507** |
| **C00-D49** | **Neoplasms** | **6025** | **4515** |
| **N00-N99** | **Diseases of the genitourinary system** | **5570** | **4926** |
| **J00-J99** | **Diseases of the respiratory system** | **5294** | **4690** |
| **M00-M99** | **Diseases of the musculoskeletal system and connective tissue** | **5018** | **4228** |
| **P00-P96** | **Certain conditions originating in the perinatal period** | **4700** | **4426** |
| **A00-B99** | **Certain infectious and parasitic diseases** | **2867** | **2536** |
| **V00-Y99** | **External causes of morbidity** | **2104** | **2053** |
| **L00-L99** | **Diseases of the skin and subcutaneous tissue** | **2023** | **1853** |
| **H00-H59** | **Diseases of the eye and adnexa** | **889** | **841** |
| **H60-H95** | **Diseases of the ear and mastoid process** | **562** | **516** |
| **U00-U85** | **Codes for special purposes** | **85** | **84** |
